# Supplementary figures and images for: Maternal smoking DNA methylation risk score associated with health outcomes in offspring of European and South Asian ancestry
Source: eLife. 2024 Aug 14;13:RP93260. doi: 10.7554/eLife.93260 (PMC11324234; doi:10.7554/eLife.93260)

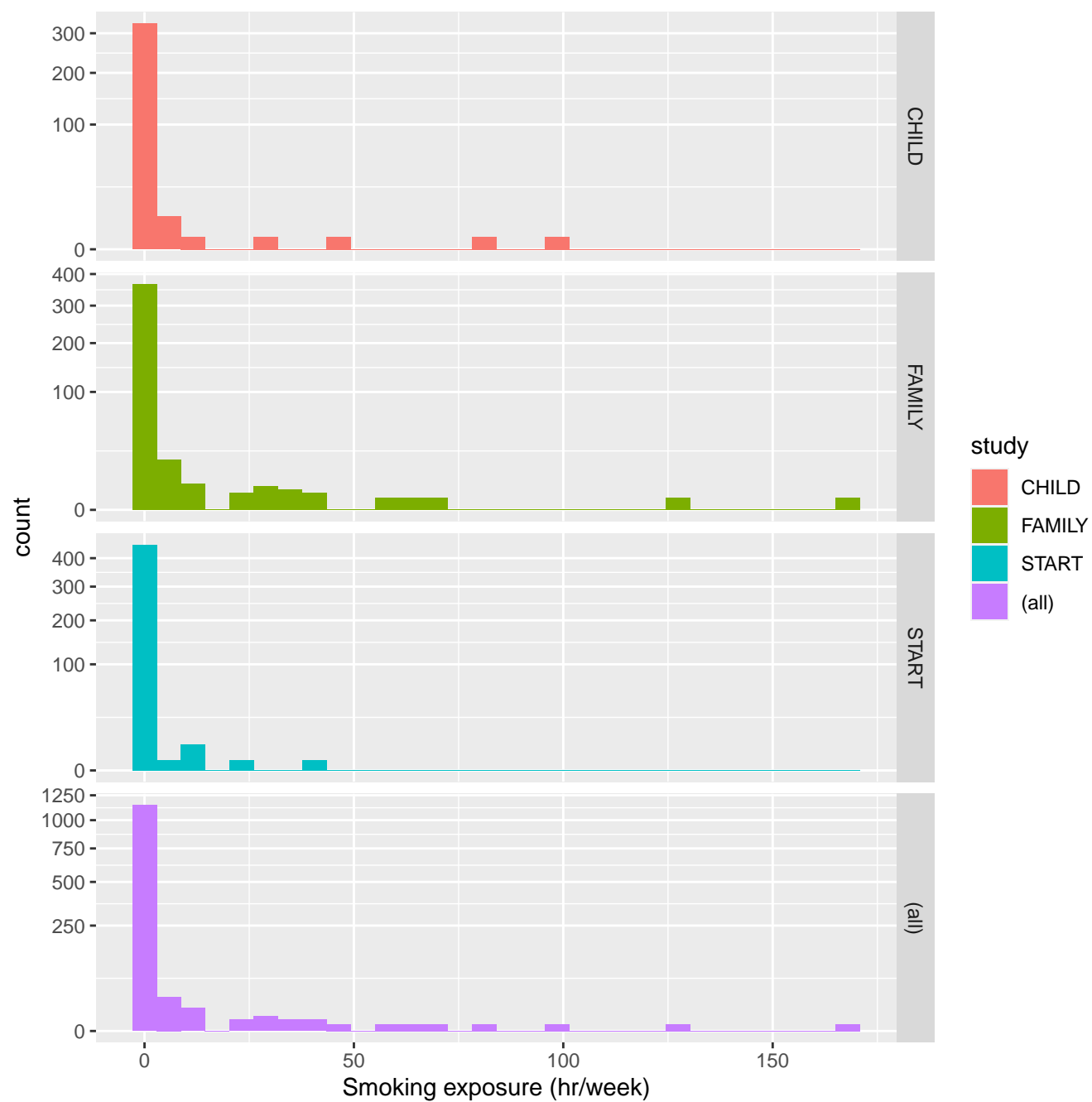

Supplement: Figure 2—source data 1. [file elife-93260-fig2-data1.pdf]
